# Neighbors-based prediction of physical function after total knee arthroplasty

**DOI:** 10.1038/s41598-021-94838-6

**Published:** 2021-08-18

**Authors:** Chong Kim, Kathryn L. Colborn, Stef van Buuren, Timothy Loar, Jennifer E. Stevens-Lapsley, Andrew J. Kittelson

**Affiliations:** 1 Department of Biostatistics and Informatics, Colorado School of Public Health, University of Colorado, Aurora, CO USA; 2 Netherlands Organization for Applied Scientific Research TNO, Leiden, The Netherlands; 3 Department of Methodology and Statistics, University of Utrecht, Utrecht, The Netherlands; 4 Physical Therapy Program, Department of Physical Medicine and Rehabilitation, University of Colorado, Aurora, CO USA; 5 Eastern Colorado VA Geriatric Research, Education, and Clinical Center (GRECC), VA Eastern Colorado Health Care System, Aurora, CO USA; 6 School of Physical Therapy and Rehabilitation Science, University of Montana, Missoula, MT USA

**Keywords:** Prognosis, Musculoskeletal system

## Abstract

The purpose of this study was to develop and test personalized predictions for functional recovery after Total Knee Arthroplasty (TKA) surgery, using a novel neighbors-based prediction approach. We used data from 397 patients with TKA to develop the prediction methodology and then tested the predictions in a temporally distinct sample of 202 patients. The Timed Up and Go (TUG) Test was used to assess physical function. Neighbors-based predictions were generated by estimating an index patient’s prognosis from the observed recovery data of previous similar patients (a.k.a., the index patient’s “matches”). Matches were determined by an adaptation of predictive mean matching. Matching characteristics included preoperative TUG time, age, sex and Body Mass Index. The optimal number of matches was determined to be m = 35, based on low bias (− 0.005 standard deviations), accurate coverage (50% of the realized observations within the 50% prediction interval), and acceptable precision (the average width of the 50% prediction interval was 2.33 s). Predictions were well-calibrated in out-of-sample testing. These predictions have the potential to inform care decisions both prior to and following TKA surgery.

## Introduction

Total Knee Arthroplasty (TKA) is the most commonly performed inpatient elective surgery in the United States, at approximately 700,000 procedures per year^1^. Although TKA is regarded as effective, the clinical course is highly variable^2^. Depending on the patient, recovery of physical function can occur within weeks, or it can be an arduous months-long process^3,4^. Moreover, the surgical population is remarkably heterogeneous. Some patients engage in sporting activities (e.g., tennis, skiing)^5^, while others struggle to ambulate at walking speeds sufficient for independence in the community. There is no such thing as the “average” patient^6^.

To achieve the ideals of person-centered care^7–9^, and also because TKA is an elective procedure, clinical decisions should be anchored to the individual patient^10^. Yet the determination of an individual patient’s functional prognosis is challenging. Prediction models have been developed in TKA, but these models have several limitations: (1) they perform poorly in out-of-sample testing^11^, (2) they are based on mathematical functions that are unlikely to be flexible enough to realistically portray the clinical course across all patients^12^, or (3) they predict functional outcomes at discrete postoperative time points, which may not overlap with the time frame during which patients are undergoing postoperative care and clinical monitoring^13^.

Neighbors-based predictions may overcome some of these limitations. In a neighbors-based approach, an index patient’s prognosis is estimated from the observed recovery data of previous similar patients^14^. These previous patients are known as the index patient’s neighbors or “matches”. In this approach, the parameters of the prediction and the shape of the prognostic trajectory are allowed to vary substantially across individuals. Such flexibility may better accommodate the heterogeneity in recovery following TKA^15^. This may also enhance the generalizability of the approach. The prediction is generated based only on a subset of patients with characteristics similar to the index patient; this contrasts with traditional prediction approaches where model parameters are heavily informed by the aggregated characteristics of the sample.

The purpose of this study was to develop and test a neighbors-based prediction approach for functional recovery after TKA surgery^16^. The outcome of interest for this analysis was the Timed Up and Go (TUG) test, a clinically feasible test of mobility and a surrogate of lower extremity strength. We utilized a combination of clinical and research data, collected longitudinally over the first six months following surgery. We divided the patients temporally (by date of surgery) into a training set and test set. This split was made to mimic how the approach would be developed and tested in clinical practice. The training set was used to tune the neighbors-based prediction, particularly to choose the optimal number of matches required to achieve optimal performance. The training set then also served as the donor dataset for an out-of-sample validation using patients from the test set.

## Methods

### Data sources

**Figure 1:**
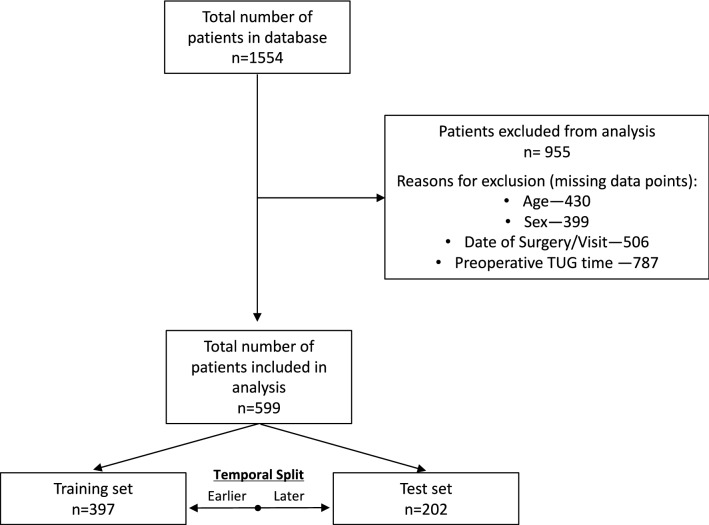
CONSORT flow diagram of patient data included in the analysis.

This analysis utilized two existing data sources involving patients with primary, unilateral TKA: (1) data collected in routine clinical practice and (2) data from previously published longitudinal research studies. Clinical data were obtained via routine quality improvement procedures at ATI physical therapy (Greenville, SC), with surgery dates between January, 2013 and June, 2017. Research data were obtained from four previously published studies, with surgery dates between June, 2006 and May, 2017. The inclusion/exclusion criteria for these research studies have been reported elsewhere^17–20^. Clinical data were not selected based on patient criteria (i.e., all patients with clinical visits were included in the dataset), although only patient records containing a preoperative and postoperative TUG assessment were utilized in this analysis. The combined dataset was divided temporally, based on surgical date, into a training set and a test set (Fig. 1). All participants provided informed consent. All records were de-identified prior to use in this study, and all methods were approved by the Colorado Multiple Institutional Review Board (COMIRB) and carried out in accordance with relevant regulations.

### Timed up and go (TUG) test

The TUG is a brief test of mobility, where a patient rises from a chair, walks a distance of 3 m and returns to a seated position in the chair. Patients were instructed to perform the test, “as quickly but as safely as possible”. The TUG demonstrates high test–retest reliability and responsiveness^12,21,22^. All testers involved with data collection for this analysis followed the same set of standardized instructions for performing the TUG test^22^.

### Matching characteristics

Variables used for selecting matches were patient factors common across all datasets: age (years), sex, Body Mass Index (BMI; kg/m^2^), and preoperative TUG time (seconds).

### Statistical analysis

All analyses were conducted using R version 3.5.1. The steps to generate a neighbors-based prediction by predictive mean matching are summarized in the following sections and also described in Supplementary Material (Box 1).

### Selection of matches by predictive mean matching

Because the source datasets contained TUG assessments at irregular postoperative time-points, we estimated a 90-day postoperative TUG time for all patients using linear mixed effects models via the brokenstick package (R statistical computing etc.)^23–25^. The 90-day time-point was used as the distal anchor for selecting matches by predictive mean matching^26^. Briefly, a brokenstick model was fit to patients in the training data with 4 knots at specific timepoints after surgery (k = 0; 14; 50; 90). Patients in the training data were then matched according to the 90-day predicted TUG time by building a linear model with matching characteristics as predictors and the 90-day brokenstick-estimated TUG time as the outcome variable.

### Flexible modeling of observed data

For each patient in the training data, the observed postoperative TUG data of the patient’s matches were used to fit a Generalized Additive Model for Location Scale and Shape (GAMLSS)^27^. The GAMLSS model was chosen for its flexibility in modeling the median (location), variance (scale), skewness and kurtosis (shape) of the TUG as a smooth function of time (i.e., time since TKA). In particular, since TUG times are positively skewed it was preferable to employ a modeling framework that accommodates flexibility in skewness over time. A cubic spline smoother with 3 degrees of freedom (df) for the location parameter and 1 df for the scale and shape parameters was employed.

### Model tuning via within-sample testing

The optimal number of matches (m) was chosen by the following procedure: (1) GAMLSS models were fit to the matches’ observed data for each of the 397 patients in the training set, with the number of matches ranging from 10 to 397 (i.e., the total number of available patients in the training data), (2) at each increment (i.e., 10 matches; 11; 12; : : : ; 397 matches), the average bias, coverage, and precision of the predictions were calculated, and (3) the optimal number of matches was determined globally by the solution that minimized bias and optimized precision whilst retaining accurate coverage (see Supplementary Material; Box 2).

### Internal and external validation

To test the performance of the predictions, we compared predicted vs. observed TUG times via calibration plots. For both the training and test sets, we binned the predicted TUG times by deciles. Within each decile of predicted data, the median and the standard error (95% Confidence Interval) of the observed data were calculated. The median was a better measure of central tendency given the skewness of the TUG data.

## Results

In the training data set we analyzed information on 397 patients with 1,339 post-operative TUG observations. We used information on 202 patients (604 observations) in the testing data. Patient characteristics from training and testing data are shown in Table 1. Although the sex distribution and BMI were similar across the two data sets, there were statistically significant differences in age and baseline TUG time. Compared to the patients in the training data, patients in the test data were approximately 2 years older on average, with 1 s slower baseline TUG times.

**Table 1:** Baseline characteristics of training and test datasets.

	Train	Test	p-value^*a*^
	(n = 397, 1339 observations)	(n = 202, 604 observations)	
Age, years; mean (sd)	64.04 (8.43)	65.90 (8.84)	0.012
Sex distribution, n (% male)	185 (46.6)	84 (41.6)	0.280
BMI, kg/m^2^; mean (sd)	31.33 (5.82)	31.98 (6.20)	0.208
Preop TUG, seconds; mean (sd)	9.98 (4.95)	11.00 (5.04)	0.018

^a^Continuous variables tested with one-way analysis of variance; Categorical variables tested with χ^2^ test.

Preop TUG, preoperative timed up and go time; sd, standard deviation; BMI, body mass index.

### Selection of matches and model tuning

#### Predictive mean matching

Age (ß = 0.037; p = 0.001), sex (ß = 0.92; p < 0.001), BMI (ß = 0.037; p = 0.02), and preoperative TUG time (ß = 0.21; p < 0.001) demonstrated a statistically significant relationship with brokenstick estimates of the 90-day post-operative TUG time. Preoperative TUG time carried the biggest weight in selecting matches; the standardized coefficient for preoperative TUG time was 4.7 times larger than for BMI.

#### Examining the optimal number matches

**Figure 2:**
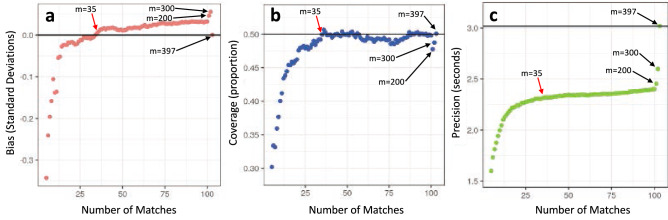
Performance metrics for neighbors-based predictions across increasing number of matches in the training dataset: (**a**) bias, (**b**) coverage, and (**c**) precision. The optimal number of matches (m = 35) is indicated with a red arrow.

**Figure 3:**
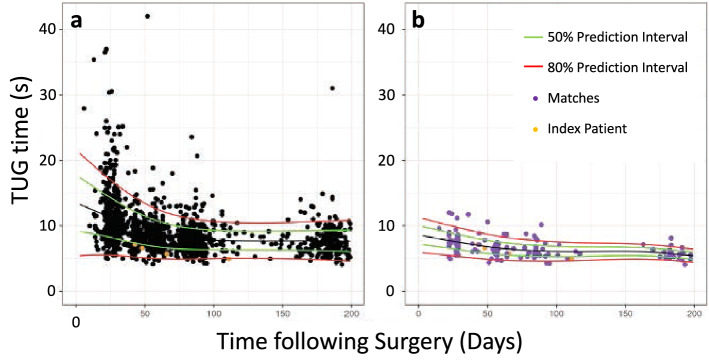
The 50% prediction interval (PI) for (**a**) the population-level estimate, is wider than the 50% prediction interval for (**b**) the neighbors-based prediction, for an example patient: a 55-year-old male with BMI of 30 kg/m^2^ and preoperative TUG time of 8 s.

The optimal number of matches was found to be m = 35 based on the low bias (0.005 standard deviations) and accurate coverage (proportion of realized observations within the 50% prediction interval: 0.50). Additionally, the average width of the 50% prediction interval with m = 35 matches was 2.33 s (Fig. 2). With m = 397 matches (i.e., the full training dataset), the average precision was 3.03 s. Thus, the neighbors-based prediction with m = 35 matches resulted in a 23% improvement in precision (Fig. 3).

### Performance via internal and external validation

**Figure 4:**
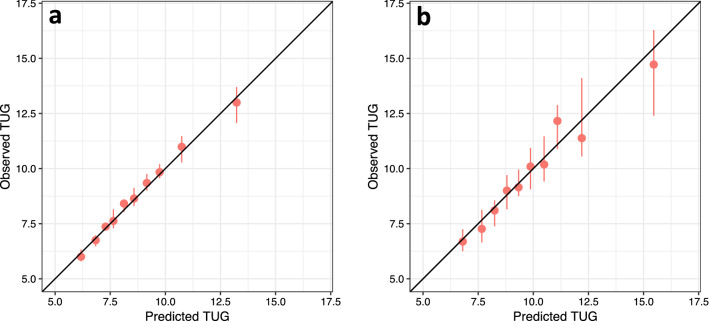
Calibration plots for neighbors-based predictions in: (**a**) training and (**b**) test datasets. Training and test datasets were divided into deciles according to the predicted TUG times. For each decile, the median observed TUG time is plotted against the median predicted TUG time. Error bars indicate the standard error of the median.

Once the number of matches was fixed via tuning procedures in the training dataset, the within-sample and out-of-sample calibration was examined. The training dataset supplied donor data for both of these analyses. This mimics how the development and testing of the approach would work in practice. Model calibration was good, with close agreement between predicted and observed values of post-operative TUG times (Fig. 4).

## Discussion

We developed and tested a novel, neighbors-based prediction for physical function following TKA. Via predictive mean matching, Body Mass Index (BMI), sex, age, and preoperative TUG time were used to identify the matches for an index patient. In our approach, the observed data from these matches were then used to generate a prediction for a new patient’s TUG prognosis. One of our primary findings was the number of matches (m = 35) required to generate predictions with optimal bias, coverage, and precision. This solution demonstrated very low bias and accurate coverage. Additionally, the 50% prediction interval was 2.33 s, on average. This amounts to a 23% improvement in precision, compared to prognostic estimates derived from the whole sample (50% prediction interval = 3.03 s).

The predictions were well-calibrated in both the training and test datasets. In a temporally distinct test sample of patients with later surgical dates, the predictions performed accurately across all deciles of observed data. This was especially encouraging given the differences in patient characteristics between training and test datasets (Table 1). Moreover, national-level changes to TKA care and reimbursement occurred during the period of data collection^28^. Such factors are likely to make external validation more challenging, but our initial analysis suggests the neighbor’s based prediction approach is at least somewhat robust. To our knowledge, this is the first study to successfully validate a prediction model for physical function in TKA.

There are several features of the approach that may have contributed to the observed accuracy of the predictions. First, estimates were based on flexible models of empirical observations, which may have allowed for more realistic representations of recovery compared to previous approaches. Second, the selection of neighbors and subsequent prediction were performed independently for each patient. Thus, each patient served as the nucleus of his or her own prediction. This may have improved external validity since each individual patient’s prediction was generated from similar patients’ observed recovery. Finally, matches were determined by an adaptation of predictive mean matching around estimated 90-day TUG times. Therefore, the matching characteristics were each weighted according to the strength of the relation to the outcome of interest. This differs from more conventional sequential *k*-nearest neighbors’ approaches, where the measure to express distances between patients is pre-set without an explicit role for the outcome of interest.

Our analysis was limited to the matching characteristics available in our source data (i.e., age, sex, BMI, and preoperative TUG time). The use of additional matching characteristics might allow for a further-refined matching strategy, resulting in improvements to the precision of the predictions. For example, patients’ pain status, comorbidity status, or surgical variables (i.e., implant type, procedure type) might be expected to influence prognosis. Future analyses that incorporate these variables would be worth pursuing. However, it is likely that some unmeasured variables are co-linear with age, sex, BMI, and preoperative TUG time and are thus somewhat baked into the current analysis. Moreover, our results suggest that the neighbors-based predictions performed well even with a small number of matching characteristics.

A limitation of our study is the use of patient data from a small number of research and clinical datasets, as care paradigms and patient demographics may differ across settings and geographical locations. Additionally, our relatively high rates of missingness may be attributed to the challenges of performing rigorous data collection in the context of routine clinical practice. Thus, our study sample is likely to differ from other specific patient samples. Our calibration findings in a temporal validation are encouraging. Nevertheless, the prediction approach should be tested in prospectively enrolled participants to further examine the generalizability.

In conclusion, a novel neighbors-based prediction approach was used to estimate postoperative TUG times following TKA surgery, utilizing patient age, sex, BMI, and preoperative TUG time. Predictions performed accurately in estimating observed TUG times at any point during first six months following surgery, according to both within-sample and out-of-sample testing. This approach could be used to inform the understanding of functional prognosis for individual patients for this common elective surgery.

## Supplementary Information

Supplementary Information 1.

Supplementary Information 2.
