# Supplementary material for: Neighbors-based prediction of physical function after total knee arthroplasty
Source: Sci Rep. 2021 Aug 18;11:16719. doi: 10.1038/s41598-021-94838-6 (PMC8373960; doi:10.1038/s41598-021-94838-6)
Supplement: Supplementary file 1 — Supplementary Information 1. [file 41598_2021_94838_MOESM1_ESM.pdf]

<u>Box 1: Neighbor's-Based Prediction via Predictive Mean Matching</u>

1. A Brokenstick model was fit to the training data to estimate a TUG observation at 90 days following surgery for all patients in the training set.
2. A multivariable linear model was fit with this 90-day estimate as the outcome and "matching characteristics" (e.g., age, sex, preoperative TUG time) as predictors.
3. The realized TUG observations from the matches were modeled with Generalized Additive Models for Location Scale and Shape (GAMLSS) to generate the neighbors based prediction.
4. A Leave One Out Cross Validation (LOOCV) approach used to identify the optimal number of matches (*m*) based on prediction performance.
5. Using the optimal *m*, patients in the testing data were matched to patients in the training data to generate neighbors-based predictions via GAMLSS.
