# Supplementary material for: Neighbors-based prediction of physical function after total knee arthroplasty
Source: Sci Rep. 2021 Aug 18;11:16719. doi: 10.1038/s41598-021-94838-6 (PMC8373960; doi:10.1038/s41598-021-94838-6)
Supplement: Supplementary file 2 — Supplementary Information 2. [file 41598_2021_94838_MOESM2_ESM.pdf]

Box 2: Assessment of Prediction Performance

1. Bias: Standardized difference in observed vs. predicted TUG times (i.e., z-score). The ideal z-score = 0.
2. Coverage: The percent of occurrences where the observed TUG time falls within the 50% prediction interval of the neighbors-based prediction. The ideal coverage is 50%.
3. Precision: The width of the 50% prediction interval. Narrower is better.
